# Discovering feature relevancy and dependency by kernel-guided probabilistic model-building evolution

**DOI:** 10.1186/s13040-017-0131-y

**Published:** 2017-03-15

**Authors:** Nestor Rodriguez, Sergio Rojas–Galeano

**Affiliations:** Universidad Distrital FJC, School of Engineering, Bogota, Colombia

**Keywords:** Relevancy discovery, Dependency estimation, Feature selection, Epistasis, Hepatitis dataset, Visual programming tools

## Abstract

**Background:**

Discovering relevant features (biomarkers) that discriminate etiologies of a disease is useful to provide biomedical researchers with candidate targets for further laboratory experimentation while saving costs; dependencies among biomarkers may suggest additional valuable information, for example, to characterize complex epistatic relationships from genetic data. The use of classifiers to guide the search for biomarkers (the so–called *wrapper* approach) has been widely studied. However, simultaneously searching for relevancy and dependencies among markers is a less explored ground.

**Results:**

We propose a new wrapper method that builds upon the discrimination power of a weighted kernel classifier to guide the search for a probabilistic model of simultaneous marginal and interacting effects. The feasibility of the method was evaluated in three empirical studies. The first one assessed its ability to discover complex epistatic effects on a large–scale testbed of generated human genetic problems; the method succeeded in 4 out of 5 of these problems while providing more accurate and expressive results than a baseline technique that also considers dependencies. The second study evaluated the performance of the method in benchmark classification tasks; in average the prediction accuracy was comparable to two other baseline techniques whilst finding smaller subsets of relevant features. The last study was aimed at discovering relevancy/dependency in a hepatitis dataset; in this regard, evidence recently reported in medical literature corroborated our findings. As a byproduct, the method was implemented and made freely available as a toolbox of software components deployed within an existing visual data–mining workbench.

**Conclusions:**

The mining advantages exhibited by the method come at the expense of a higher computational complexity, posing interesting algorithmic challenges regarding its applicability to large–scale datasets. Extending the probabilistic assumptions of the method to continuous distributions and higher–degree interactions is also appealing. As a final remark, we advocate broadening the use of visual graphical software tools as they enable biodata researchers to focus on experiment design, visualisation and data analysis rather than on refining their scripting programming skills.

**Electronic supplementary material:**

The online version of this article (doi:10.1186/s13040-017-0131-y) contains supplementary material, which is available to authorized users.

## Background

**Fig. 1:**
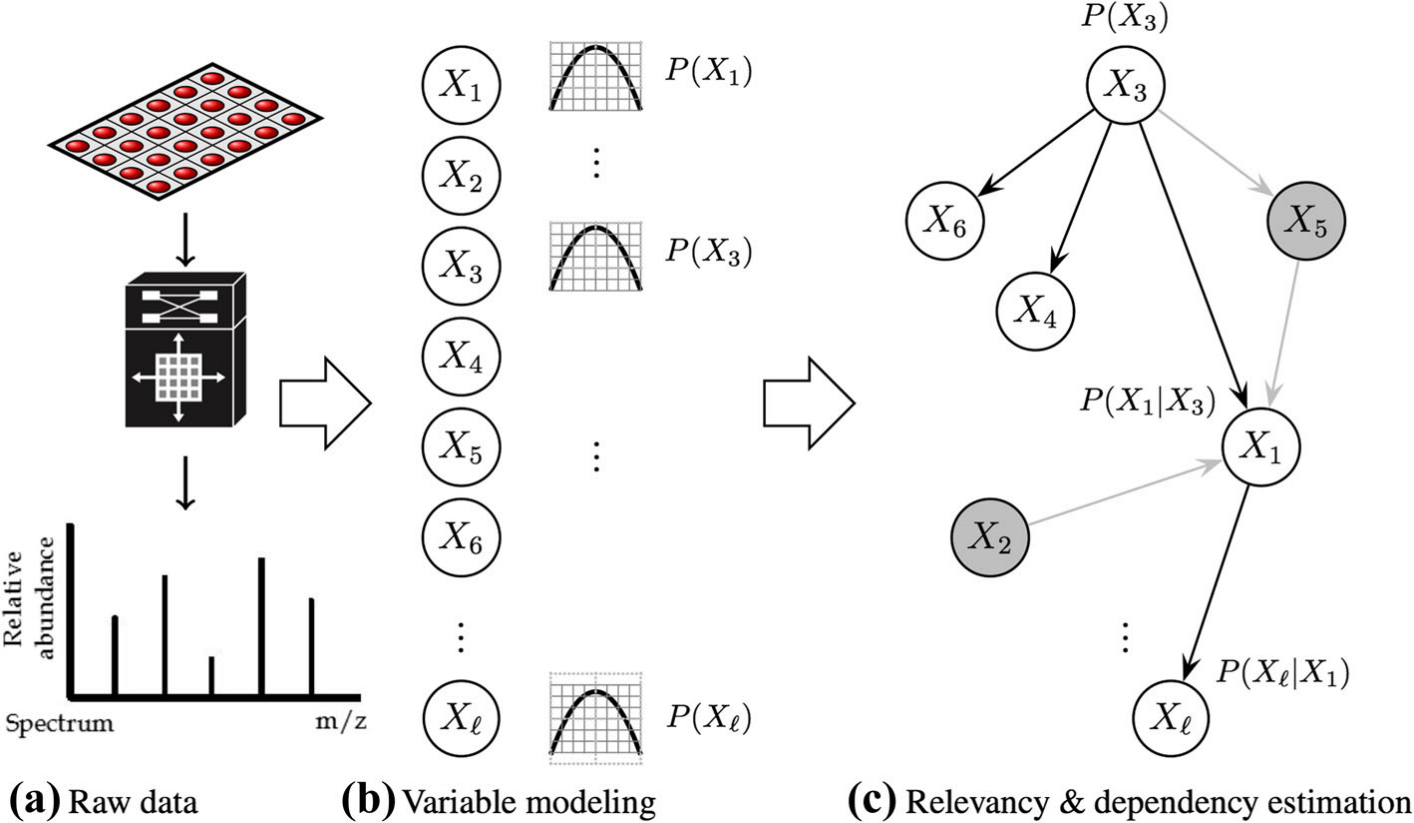
Depiction of simultaneous variable selection and dependency estimation in data mining. **a** A dataset built with any data acquisition technology (here illustrated as a schematic high–throughput proteomic analysis of blood samples in a protein array using a mass spectrometer). **b** Marginal effects (independence) of the variables describing the data are modeled. **c** Interacting effects (dependencies) among variables are estimated, relevant variables are selected, whilst irrelevant variables along with associated dependencies are discarded (shown in *grey*)

The current state of data acquisition technology is providing industry and academy with large–scale sources of information that are relatively cheap to store and collect, but expensive to process and understand; this phenomenon is pervasive to domains as diverse as bioinformatics, fraud detection, computer vision, recommender systems, particle physics, financial analysis, weather forecasting, and social networks media streaming, to name a few. One of the main challenges arising in processing such huge amounts of data, is to discover from the many observed variables (also known as *features*), those that are most relevant to explain significant patterns –or markers– of hidden concepts. This task is known as feature selection in the data–mining community or biomarker discovery in the biomedical ambit. In addition to relevancy discovery, also of interest is the identification of interactions (dependencies) between those markers; a schematic depiction of such relevancy–dependency scenario is illustrated in Fig. 1.

Relevancy estimation techniques are aimed at finding subsets of markers in datasets with a high number of dimensions, where noisy, redundant or irrelevant variables abound. The selected features may become targets of more detailed studies requiring expensive experimentation or human expertise, thus saving costs and time not spent on the discarded variables. This problem of selecting the relevant variables can be regarded as a search procedure over the space of all possible combinations of variable subsets, therefore, an NP-Hard problem [1]; similarly, finding the underlying structure of a graph representing dependencies between those variables is also combinatorial [2]. Thus the need of using approximating, iterative methods is an alternative to find suitable solutions.

Research in techniques for discovering relevant variables is a very active field in the data mining community (see e.g. [2–7]), and has attracted much attention in the last two decades [8]. The *filter* approach ranks the variables according to a linear criterion such as their correlation to the prediction target; they are computational simple, but usually fails to capture non–linear patterns of discrimination.

In contrast, the *wrapper* approach performs the search guided by the classification accuracy of a discriminant rule that evaluates the suitability of a subset of features; although computationally more demanding, this setting is able to find smaller and more discriminative sets of relevant features, specially when non–linear concepts are hidden. In this respect several approaches have been proposed previously using different metaheuristics, probabilistic assumptions and discrimination techniques. One particular flavor uses probabilistic model–building genetic algorithms combined with well–known classifiers (a review of applications of this approach in the bioinformatics domain can be found in [9]). This kind of algorithms simultaneously estimate the parameters of a probabilistic relevance model of the variables *and* the structure of a graph representing relationships among them. Recent studies using a Bayes network as such model have reported promising results in discovering interactions in genetic data [2, 6, 10, 11].

In a similar vein, here we describe a novel method that models relevancy and dependency by coupling a weighted kernel machine for pattern classification [12] into a probabilistic–based genetic algorithm [13] for dependency estimation. Previous studies considered combining classical and probabilistic genetic algorithms with weighted kernel classifiers for relevancy–only discovery [14, 15]; our contribution in this paper is to extend those approaches to take advantage of the discrimination power of a weighted kernel classifier to guide the search for a probabilistic model that simultaneously estimates marginal and interacting effects among the features in a discrimination problem.

## Method

### Previous work

#### Overview of probabilistic-based genetic algorithms

This kind of genetic algorithms are stochastic search techniques that evolve a probability distribution model from a pool of solution candidates, rather than evolving the pool itself. The distribution is adjusted iteratively with the most promising (sub-optimal) solutions until convergence. Hence, they are also known as Estimation of Distribution Algorithms (EDA, for short). The generic estimation procedure is shown in Algorithm 1. Step (1) initialises the model parameters ***θ***. Step (2) is the loop that updates the parameters ***θ*** until convergence. Step (3) samples a pool $\mathcal {S}$ of *n* candidates from the model. Step (4) ranks the pool according to a cost function *f*(·) and chooses the top-ranked into $\mathcal {B}$. Step (5) re-estimates the parameters ***θ*** from this subset of promising solutions.



The actual realisation of each step in the generic template of Algorithm 1 defines different types of EDA s: discrete or continuous parameters; binomial, multinomial or Gaussian distributions; univariate, bivariate or multivariate dependencies, see among others: [14, 16–23]. Our approach to the problem of interest is based on the following assumptions: discrete parameters, Gaussian distributions, bivariate dependencies.

**Fig. 2:**
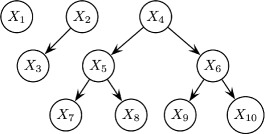
A probability model representing dependencies using a *MSF*. Here, *k*=3 connected components are depicted, where *ℓ*_1_=1,*ℓ*_2_=2,*ℓ*_3_=7 and $\ell =\sum _{k} \ell _{k}=10$ Feature order is arbitrary

For this purpose we resorted to build upon the Bivariate Marginal Estimation of Distribution Algorithm (BMDA) proposed by [19]. In BMDA the nodes of a graph ${\mathcal G}$ are associated to the problem variables *X*_*i*_, and pair-wise dependencies are represented with a *minimum-spanning-forest*, *MSF* (see Fig. 2). The roots of the *MSF* correspond to independent variables associated with marginal effects, whereas the rest of the nodes correspond to dependant variables associated to interaction effects with respect to their parents (notice that in a forest, any non–root node has at most one parent). Therefore, the probability model assumed by BMDA is the following bivariate binomial distribution with parameters ***θ***={*MSF*,{*ρ*_*i*_},{*ρ*_*ij*_}}, where **R**_*MSF*_ is the set of root variables, **E**_*MSF*_ is the set of interactions among the variables, and {*ρ*_*i*_} and {*ρ*_*ij*_} are the parameters of the independent and interacting factors respectively, of the overall probability distribution:

$$\begin{array}{*{20}l} P(X; \boldsymbol{\theta})&= \prod_{i \in \mathbf{R}_{\mathsf{MSF}}} P(X_{i}; \rho_{i}) \prod_{(i,j) \in \mathbf{{\mathbf E}_{\mathsf{MSF}}}} P(X_{i} | X_{j}; \rho_{j}, \rho_{ij})   \end{array} $$

BMDA adheres to the generic EDA template of Algorithm 1, with tailored sampling and estimation rules that we describe next for illustration purposes. Firstly, the sampling mechanism (Step (3) in the algorithm) is modified as to preserve the most promising candidates $\mathcal {B}$ from the previous iteration along with new candidates sampled from the current model ($\frac {n}{2}$ candidates):

1 $$\begin{array}{*{20}l} \mathcal{S} = \mathsf{sample}\left(P(X;\boldsymbol{\theta}),\frac{n}{2}\right) \cup \mathcal{B} \end{array} $$

Secondly, the estimation strategy (Step (5) in the algorithm) comprises the following operations:

1. Build a *disconnected* graph ${\mathcal G}({\mathbf {V}}, {\mathbf {E}}_{t})$, with **V** the set of problem variables, and **E**_*t*_ the set of variable interactions determined by a bivariate Pearson *χ* ^2^ dependency test criterion: ${\mathbf {E}}_{t} = \{(i, j) \in {\mathbf {V}} \times {\mathbf {V}} : i \neq j \land \chi _{ij}^{2} \geq 3.84\}$. Here the statistic *χ* ^2^ is computed from the current candidate pool $\mathcal {B}$ at iteration *t*.
2. Compute *MSF*(**E**_*t*_) representing variable dependencies. Build the set of root nodes **R**_*MSF*_ by choosing at random one node of every component in *MSF*(**E**_*t*_).
3. Estimate parameters {*ρ*_*i*_:*i*∈**R**_*MSF*_} and {*ρ*_*ij*_:(*i*,*j*)∈**E**_*MSF*_} using frequentist updates (see Eq. (2)), again over the current candidate pool $\mathcal {B}$ at iteration *t* (*N.B.* Here, [*c*]=1 if the argument *c* is true or 0 otherwise).
  2 $$\begin{array}{*{20}l} \rho_{i}^{a} = \sum_{k = 1}^{n} \left[\mathcal{B}_{ki} = a\right],\quad \rho_{ij}^{ab} =\sum_{k = 1}^{n} \left[\mathcal{B}_{ki} = a \land \mathcal{B}_{kj} = b\right],  \end{array} $$

#### Overview of kernel machines for pattern classification

As it was mentioned earlier, we use a classifier that guides the search for more suitable subsets of features (Step 4 of Algorithm 1). From the many pattern classification techniques, kernel machines [12] have shown outstanding performance on diverse problem domains; thus we chose them as base classifiers for our method.

Kernel machines classify patterns using a linear combination of nonlinear mappings, known as kernel functions, evaluated on the current input instance *i* over the observed instances in the past *j*<*i*, using the rule $\hat {y}_{i} = \mathsf {sign}\left (\sum _{j=1}^{i-1} \alpha _{j} \kappa ({\mathbf x}_{j},{\mathbf x}_{i})\right)$, where $\hat {y}_{i}$ is the class prediction and the coefficients {*α*_*j*_} are learnt with standard linear discriminant algorithms such as the Perceptron or the SVM [12].

**Fig. 3:**
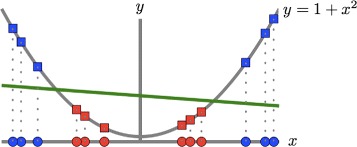
Transforming the input space onto a higher dimensional space using a nonlinear mapping *Φ*(·), may resolve nonlinearities with linear discriminants. Here a binary dataset (positive class=red, negative=blue) is visualised in two different subspaces, a line in $\mathbb {R}^{1}$ and a parabola in $\mathbb {R}^{2}$. The original data (ovals) is not linearly separable in $\mathbb {R}^{1}$. The transformed data (squares) is separable in $\mathbb {R}^{2}$ by an arbitrary linear discriminant (green). The mapping used was *Φ*(*x*)↦(*x*,1+*x* ^2^)

The classification power of kernel machines is partly due to the ability of the kernel function to map the input space into a higher–dimensional space [24], that is, *κ*(**x**_*j*_,**x**_*i*_)=*Φ*(**x**_*j*_)^⊤^ *Φ*(**x**_*i*_). In such transformed space nonlinearities are probably easier to resolve, while the classifier preserves the computational simplicity of a linear discriminant in input space [25] (see Fig. 3). There is no need to explicitly declare the nonlinear mapping as long as the kernel function is defined as a symmetric positive semidefinite function [12, 24]. Two of such widely–used kernel functions are the RBF and the polynomial kernel, $\kappa _{\sigma }({\mathbf x},{\mathbf z}) = \mathsf {exp}\left (-\sigma \sum _{i=1}^{\ell } (x_{i}-z_{i})^{2} \right)$, and *κ*_*d*_(**x**,**z**)=(**x** ^⊤^ **z**)^*d*^. The parameters *σ* and *d* define the width of the RBF and the degree of the polynomial kernel, respectively.

Modified weighted versions of these kernels incorporate scale factors **w**={*w*_1_,…,*w*_*ℓ*_:*w*_*i*_∈ [ 0,1]} for each of the *ℓ* dimensions (i.e. variables) in order to modulate their contribution to the total computation [14, 26, 27]. The weighted RBF and weighted polynomial kernels are then defined as $\kappa _{\sigma }({\mathbf x},{\mathbf z};{\mathbf w}) = \mathsf {exp}\left (-\sigma \sum _{i=1}^{\ell } w_{i}(x_{i}-z_{i})^{2} \right)$, and $\kappa _{d}({\mathbf x},{\mathbf z};{\mathbf w}) = \left (\sum _{i=1}^{\ell } w_{i}(x_{i} \cdot z_{i}) \right)^{d}$, respectively. Here we remark that as *w*_*i*_ is closer to 1, its associated variable becomes more relevant since it contributes a larger magnitude to the final value of the kernel computation. It is in this sense that we interpret the vector **w** as representing the relevancy distribution of the variables for the purposes of classification. Accordingly, classification performance will guide the EDA to estimate these relevancy factors.

Additionally we observe that because of the additive nature of these kernel functions, the resulting scaling in each dimension can be obtained by preprocessing the input data with a modified version of the weight vector **w**. For instance, regarding the RBF kernel it can be seen that:

$$\begin{array}{*{20}l} \kappa_{\sigma}\left({\mathbf x},{\mathbf z};{\mathbf w}\right) &= \mathsf{exp}\left(-\sigma \sum_{i=1}^{\ell} w_{i}\left(x_{i}-z_{i}\right)^{2} \right)= \mathsf{exp}\left(-\sigma \sum_{i=1}^{\ell} \left(\sqrt{w_{i}}x_{i}-\sqrt{w_{i}}z_{i}\right)^{2} \right) \\ &= \kappa_{\sigma}\left(\widetilde{\mathbf w} \otimes {\mathbf{x}}, \widetilde{\mathbf{w}} \otimes {\mathbf{z}}\right), \end{array} $$

where $\widetilde {{\mathbf w}} = \{\sqrt {w_{1}},\ldots,\sqrt {w_{\ell }}\}$ and ⊗ denotes the component–wise product. The case of the weighted polynomial kernel is analogous. This observation was originally pointed out in [14] and more recently in [27].

#### Related methods for relevancy and dependency estimation

Some previous studies have considered a multi-objective approach for simultaneous optimisation of accuracy and relevance distribution. Two representative techniques utilise EDA s to estimate the parameters of a probability model from which the relevance factors are sampled. One of such approaches, the EBNA algorithm [28]) uses a multivariate probabilistic model that incorporates second–order dependencies between the variables. The search of relevancy and dependencies is guided by the discriminatory power of a Naive-Bayes classifier. The authors report promising results in finding suitable variable subsets with good generalisation performance, although the benefit of obtaining insights about relationships between variables is traded–off with an overhead in computational complexity.

On the other hand, wKiera is a wrapper approach for feature relevance estimation that combines EDA s and kernel machines [14]. The estimation is carried out using an array of scale factors coupled to a weighted kernel machine whose classification accuracy guides the search for the relevance distribution using an UMDA algorithm [13]. The authors reported encouraging results compared to filter methods in discovering relevant variables on a number of different classification tasks, including problems with linear and non-linear hidden concepts in very–high dimensional spaces. The algorithm however, does not retrieve additional information about the interactions between the relevant variables, because it assumes they are conditionally independent. In this respect, wKiera differs from the method we propose in this paper which, despite combining also a kernel machine with an EDA, estimates relevance based on a probabilistic model of bivariate interactions, obtaining a network of dependencies that may provide additional insights regarding the combined effects of related features, as we shall explain in the next section.

Lastly, another well-known approach to treat dependencies is ReliefF [29]. This technique has been used to estimate feature quality in prediction and regression tasks. It can be applied as a previous step (*filter*) to feature subset selection. In contrast to other filter techniques assuming conditional independence of the features (i.e. correlation coefficient, information gain or Gini index), ReliefF detects local context interactions between variables and use that information during estimation of their relevancy. In this way, it is able to analyse combined effects due to dependencies among relevant features.

The scores computed by ReliefF are positive for relevant features and negative for irrelevant ones. Although it does not provide explicit information about the dependencies, this technique has proven fast and effective for relevance estimation on problems with strong feature interactions, where other filters become myopic and fail to find them [30].

### Proposed algorithm

The new method that we termed *weigthed Kernel Iterative Estimation of Dependencies and Relevancy Algorithm* (Kiedra for short) uses a hybridised version of BMDA and wKiera to estimate the relevancy distribution and second order dependencies of input variables. The search is guided by the suitability of the relevance factors when classifying the data with its corresponding weighted kernel SVM. The following steps were introduced in the design of Kiedra:

- When building the correlation graph, the Mutual Information (MI) criterion [31] was additionally considered to estimate dependencies between arbitrary pairs of variables, that is, to the extent to which they share information. A third Combined Mutual Information and *p*-value (SIM) criterion was also considered; the latter mixes both statistical and information–theory dependence [32]. Consequently, the rule to compute the edges on the dependency network was modified to that in Eq. (3):
  3 $$ {\mathbf{E}}_{t} = \left\{(i, j) \in {\mathbf{V}} \times {\mathbf{V}} : i \neq j \land \mathsf{any\_of} \left(\left\{\chi^{2}_{ij} \geq 3.84, \mathsf{MI}_{ij} > 0, \mathsf{SIM}_{ij} > 0\right\}\right)\right\}  $$
- When choosing the root nodes **R**_*MSF*_ of the dependency network (forest), instead of selecting at random we introduced another information–theory criterion that selects nodes minimising the marginal entropy *H*(·) in each connected component **V**_*k*_ of the network, $\bigcup _{k} {\mathbf V}_{k} = {\mathbf V} ~\land ~ \bigcap _{k} {\mathbf V}_{k}\ = \emptyset $, as stated in Eq. (4). The marginal entropy is computed frequentist-wise from the current candidate pool $\mathcal {B}$ at iteration *t*. The rationale behind the introduction of this criterion is that those nodes with lowest entropy are richer in information content, and thus good candidates to become independent parents of the dependency subnetworks (in this sense this criterion was originally proposed in the MIMIC algorithm [17]).
  4 $$ {\mathbf R}_{\mathsf{MSF}} =\left\{r_{k}: r_{k} = \underset{i}{\arg\min}\ H\!\left(X_{i} \in {\mathbf V}_{k}\right)\right\}  $$
- Finally, candidate relevance factors are sampled from the current probability model and incorporated to a population including the previous best solutions found: $\mathcal {S} \gets \mathsf {sample}(P(X;\boldsymbol {\theta }),\frac {n}{2})\cup \mathcal {B}$. Each candidate in this population ${\mathbf w}_{k} \in \mathcal {S}$ is assessed by building a weighted SVM and obtaining its classification accuracy with a 5–fold cross–validation on the modified dataset $\widetilde {D} \gets D \otimes {\mathbf w}_{k}$. The population is ranked by best accuracies and the top candidates are selected. These candidates are then used to re–estimate the dependency network and the relevance parameters of the probabilistic model, and the process iterates until these parameters converge. Further details and specification of Kiedra are given in the Additional file 1: Additional Methods and Tools section.

## Empirical study

In this section we report the results of a number of experiments designed to validate the feasibility of the proposed method. Initially we provide details about our implementation platform. Then we describe the first experiment aimed at testing the ability of the method in discovering epistasis on generated human genetic datasets; there we used ReliefF as a baseline to compare, for it is also a method that treats feature interactions. Our empirical study continued with a second experiment designed to compare the proposed approach with other EDA-based and kernel-based methods on some benchmark classification problems. Lastly, we conducted a third experiment intended again to discover relevancy and dependencies in a medical domain, specifically on a hepatitis dataset, this time corroborating the results with recent findings in the literature of that disease.

### Implementation

**Fig. 4:**
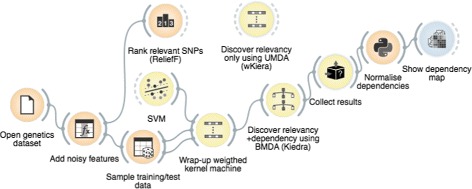
The visual program implementing Kiedra in the Orange/ Goldenberry workbench. Widgets on the left–hand side of the canvas provide the required input (data sampling, kernel machine setup, etc.) for the central *discoverer* component (Kiedra) which executes the algorithm so as to estimate relevancy and dependency, and then provides these results as inputs for the collection and visualisation widgets on the right–hand side of the canvas. Two additional widgets shown on the top of the canvas were used to implement wKiera and to execute ReliefF. See text for further details

The method was implemented using the Goldenberry suite of visual components for stochastic–based search optimisation within the Orange multi–platform workbench for data mining [33, 34]. In this environment, visual components (known as *widgets*) executing different steps of the algorithm such as data input and sampling, SVM s training, BMDA estimation, etc., are dragged onto a visual canvas where they are assembled to create the Kiedra program shown in Fig. 4. The WrapperCostFunction widget is the core of the program; it gets input from the Data and SVM widgets and wraps them up in a weighted kernel machine which in turn is provided as the cost function required by the BMDA optimiser. Additional widgets were used for comparison with ReliefF and wKiera, and also for results collection and visualisation, namely Rank, DistanceMap, BlackBoxTester and UMDA (we note in passing that the wKiera algorithm can be implemented by simply replacing BMDA with UMDA in this assemblage). More information of these widgets and their configuration can be found in the Additional file 1: Additional Methods and Tools section.

### Experiment 1: Relevancy and dependency in genetic epistasis

Genetic epistasis refers to those complex gene-gene interactions that may trigger susceptibility to a common human disease. Instead of characterising a single nucleotide polymorphism (SNP) as an isolated marker of a disease, epistasis assumes a combination of markers is in fact associated to the phenotypic manifestation of the disease. Hence, epistasis in genetic datasets is an interesting target for simultaneous relevance and dependencies mining.

In this first set of experiments we focused on evaluating the effectiveness of the method in discovering such complex interactions. For this purpose we considered a recently proposed testbed of human genetic–like model–free datasets simulating etiologies between combinations of SNPs [35]; the datasets were designed to minimise predictiveness of single or pairs of genetic variations and maximise highe–order interactions. The original datasets consisted of 3, 4 or 5 SNPs; we modified them by adding both 5 and 10 features unrelated to disease status so as to represent three, four, or five-way epistatic problems polluted with noise. The irrelevant features were sampled from an uniform random distribution as $R_{i} \sim {\mathcal {U}}(0,10)$. A summarised description of these datasets is given in Table 1; those labeled as “NoLow” indicates that no lower–effects can be found, that is, epistasis involves strong interactions among the entire set of relevant SNPs. Besides, we chose ReliefF as a baseline to compare the performance of the proposed method, considering its ability to also treat feature interactions, is well-known [30].

**Table 1:** Description of simulated epistatic problems (see [35] for further details)

Problem	Datasets	Simulated SNPs	Noisy SNPs	Disease status	Instances
3way	100	3	5 and 10	0 or 1	3000 (balanced)
4way	100	4	5 and 10	0 or 1	3000 (balanced)
4way-NL	100	4	5 and 10	0 or 1	3000 (balanced)
5way	100	5	5 and 10	0 or 1	3000 (balanced)
5way-NL	100	5	5 and 10	0 or 1	3000 (balanced)

For each dataset, the experiment was conducted following the scheme shown in Fig. 4. On the one hand, once the noise was added, features in the polluted dataset were scored using ReliefF; those obtaining positive scores were labeled as relevant, otherwise as irrelevant. On the other hand, Kiedra experiments were executed as follows. Firstly the modified dataset was split in training and testing subsets (25%/75%); then an SVM was parameterised (*C*=100, RBF kernel with *σ*=10) and wrapped–up in a weighted kernel machine along with the data. The latter was then provided as the cost function to evolve a BMDA (*n*=20,*i* *t* *e* *r*=80, SIM criterion). In view of the stochastic nature of Kiedra, the above protocol was repeated 30 times with the resulting scale factors being collected and averaged; then a cut–off threshold of 0.7 was applied to select relevant from irrelevant features.

**Fig. 5:**
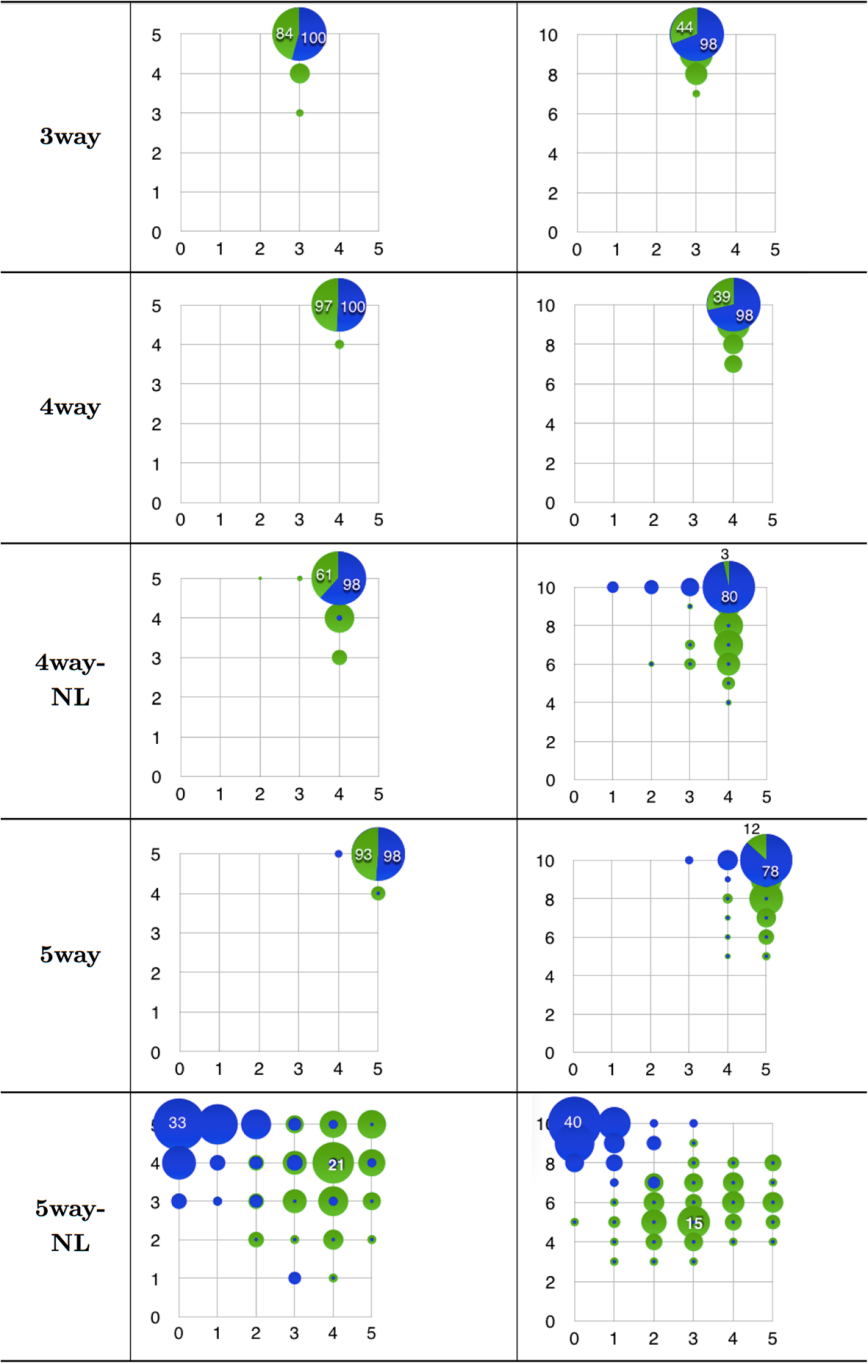
Bubble frequency plot of epistasis discovery with Kiedra (*blue*) and ReliefF (*green*). Plots are arranged by epistatic problem in rows, i.e. number of relevant SNPs, and noise pollution in columns, i.e. number of irrelevant features: 5 (*left*) or 10 (*right*). One hundred datasets per problem were analysed; a bubble in any plot depicts the relative frequency of correct discovered (#relevant, #noisy) features corresponding to its coordinate (abscissas are #relevant). Largest bubbles are the most frequent result found by Kiedra in each problem; the slices shown in such bubbles illustrate in proportion (hits) how often ReliefF agreed with that finding

The subsets found with both methods were finally contrasted to the ground truth in order to record a coordinate (*#* *R*,*#* *N*) of the correct number of relevant (*#* *R*) and noisy (*#* *N*) features that were discovered. These records are summarised in the bubble frequency plots of Fig. 5. They are arranged according to their pollution rate (5 or 10 added noisy features). Here, the area of any bubble in each problem represent the frequency at which the method hit the corresponding coordinate (*#* *R*,*#* *N*) of relevant and noisy features within the 100 datasets.

Let us examine first the plots in the left-hand column of the figure. In the first two problems, 3way and 4way, Kiedra was able to discover the correct number of relevant and noisy features within the whole collection of datasets. Likewise, in problems 4way-NL and 5way, Kiedra only missed a few 2 datasets in each problem. ReliefF in turn, discovered correctly up to 84, 97 and 93 datasets in problems 3way, 4way and 5way, achieving a lower rate of 61 hits for problem 4way-NL. These results hint at the ability of Kiedra to discover epistatic effects even with coexisting uninformative markers. ReliefF shows a comparable trend, except in problem 4way-NL where probably the higher–order dependencies causes some trouble so as to find the correct relevant features.

Now, let us discuss the results shown in the plots of the right–hand column of the figure. We recall that these problems were modified with twice the number of polluted features. A similar trend can be seen for the behaviour of Kiedra. The method achieved a correct hit rate of 98 out of 100 in problems 3way and 4way; this rate was down to 80 in problem 4way-NL and slightly lower in 5way. On the other hand, ReliefF was adversely affected by such level of noise, for it obtained correct hit rates of 44, 39, 3 and 12 respectively.

Finally, let us comment on the plots of the last row in the figure (problem 5way-NL), whose results differ amply from those reported previously. In this problem, ReliefF seems to perform better in comparison with Kiedra, although not a conclusive trend is evident. In the 5-noisy features problem it is able to find the correct coordinate (5,5) in about 10 hits, but the most frequent result was coordinate (4,4) with 21 datasets. Likewise in the 10-noisy features problem the most frequent discovered coordinate was (3,5) while the optimal (5,10) was never found. On the other hand, Kiedra clearly underperformed in this problem as their findings are highly biased to coordinates where the correct number of noisy features are identified, but in contrast few or none of the relevant are found. We recall that the originators of these epistatic datasets reckon that this is the hardest problem, as its etiology comprises interactions of the entire set of 5 SNPs, while no lower-degree interactions were enabled, as opposed to the other problems [35]. We remark however, that the dependency graph in which Kiedra is based assumes a bi–variate probability distribution, which may explain why it fails in modeling higher–order interactions appropriately.

**Fig. 6:**
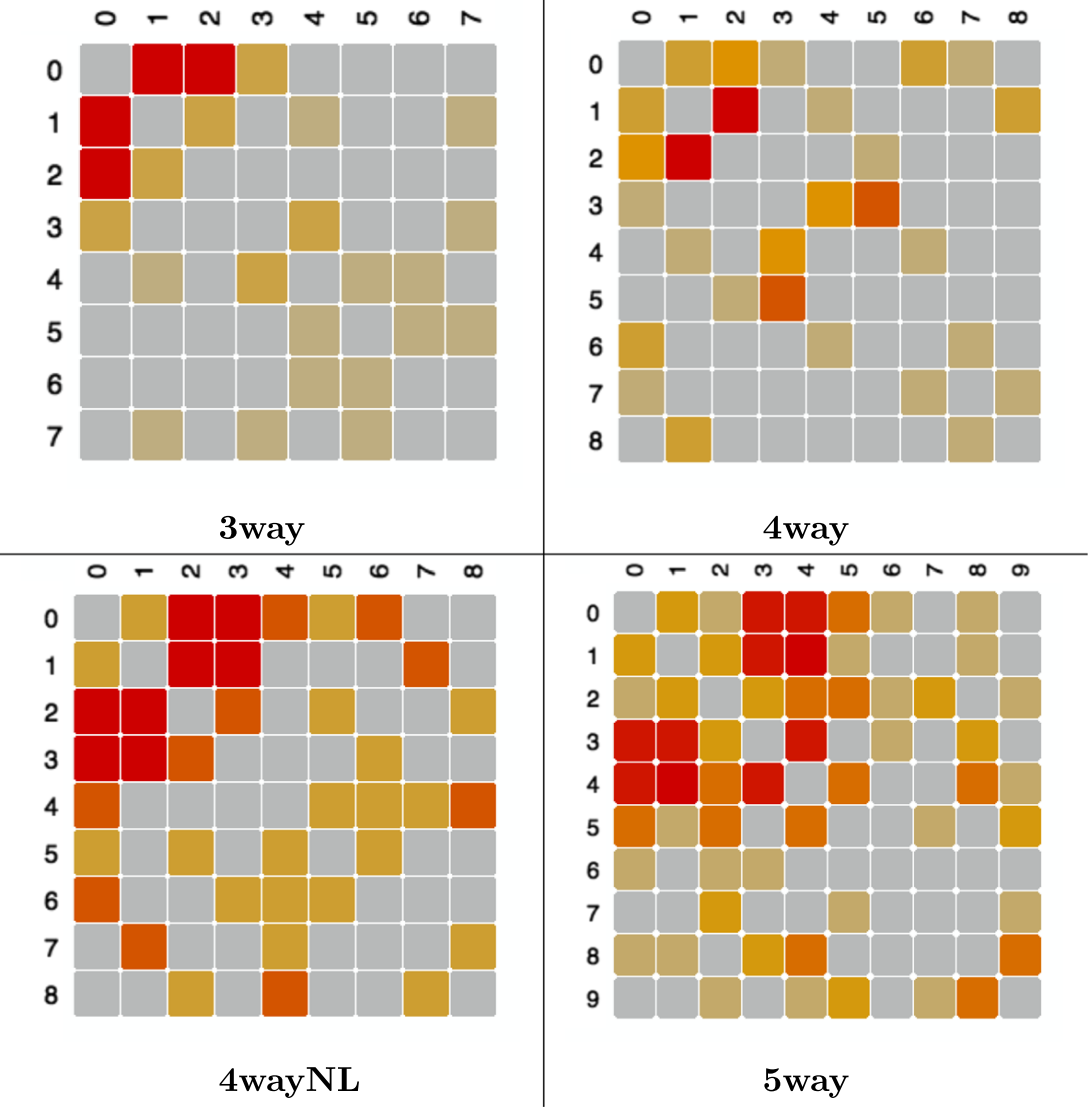
Dependency heat maps in epistatic problems with 5 added noisy features. These matrices represent dependency graphs (gene–gene interactions) computed by Kiedra (notice that dependencies are non–directed since matrices are symmetric). Relevant variables are numbered starting from 0 (i.e. {0,1,2} in problem 3way, {0,1,2,3} in problem 4way and so on). Interaction intensity is represented by a warm–based gradient palette

On a different note, it is worth mentioning that at the expense of obtaining explicit information about gene–gene interactions, Kiedra is computationally more demanding than ReliefF. This is because Kiedra requires training and testing a classifier at every iteration during the evolution of its probability model. This effort is compensated however, by its ability to explicitly compute the feature dependency graph while searching for the relevant variables. To illustrate this point, Fig. 6 shows examples of dependency heatmaps generated from the dependency graph computed by Kiedra for arbitrary chosen datasets belonging to problems 3way to 5way (5 noisy features), whose epistasis, as it was discussed above, was correctly discovered by the method. These dependency heatmaps are symmetric matrices that were visualised using the ShowDependencyMap widget of Fig. 4. Notice that each interaction map is meant to be interpreted jointly with the associated relevance factors, in order to identify informative interactions between relevant features and to ignore irrelevant interactions. For example, in the shown heatmaps the epistatic interactions would be located in the section of the matrices involving features 0 to 2 (3way) or 0 to 3 (4way) or 0 to 4 (5way), as those were the features correctly selected as relevant by Kiedra. In contrast, one can argue that the remainder sections of the matrices contain spurious dependencies arising from correlations due to randomness of the added noise, a fact corroborated because these features were correctly designated as irrelevant by the method.

### Experiment 2: Feature relevance discovery in benchmark classification problems

This second set of experiments were conducted to study the relevance discovery ability of Kiedra on classification problems, in comparison with the other EDA–based and kernel–based techniques described in Section 1. We chose a benchmark of five datasets from the UCI repository [36] (see description in Table 2). The results for EBNA were taken from those reported in [28]. wKiera and Kiedra were implemented with the visual components of Fig. 4.

**Table 2:** Description of benchmark classification datasets

Dataset	Variables	Classes	Instances
Ionosphere	34	2	351
Soybean	35	19	307
Horse–colic	27	24	368
Annealing	38	6	798
Image	19	7	2310

One experiment was conducted per each dataset as follows. The dataset was initially preprocessed as to fill–in missing values with a Naive Bayes classifier and to normalise within a [ 0,1] real interval. The processed dataset was then randomly split into *training* and *test* subsets of equal size. These subsets are the inputs for the cross–validation scheme used to estimate the accuracy of each candidate solution. For each method, 10 repetitions were executed with different random splits. Average statistics were collected using the BlackBoxTester widget.

We evaluated the performance of these methods in two aspects: relevancy discovery and classification accuracy. Firstly, let us examine the average number of relevant variables per dataset, which are reported in Table 3. It is clear that Kiedra and wKiera show comparable results; besides both obtained smaller variable subsets than those of EBNA. Kiedra was able to outperform wKiera in three cases. Reduction rates in the number of variables with respect to the original dimensionality, varied from around 76% or more (Ionosphere, Soybean and Annealing) to 60% (Image) to 40%, (Horse–colic). It is worth noting that in two cases (Ionosphere and Annealing), the new method achieved around half the size of the subsets found with EBNA; moreover, these were among the datasets with higher number of raw variables.

**Table 3:** Average number of relevant variables discovered in each dataset

Dataset	Raw	EBNA	wKiera	Kiedra
Ionosphere	34	13.40±2.11	7.30±0.82	7.20±0.92
Soybean	35	6.10±1.85	5.10±0.99	6.50±1.58
Horse–colic	27	18.90±2.76	16.40±1.90	16.60±2.12
Annealing	38	20.50±3.13	9.60±0.97	9.40±1.43
Image	19	8.00±0.66	7.72±1.24	7.45±1.36

Regarding average classification accuracy (see Table 4), again Kiedra and wKiera show similar performance, with a slight advantage to Kiedra in two datasets. The similarity in the performance of these two techniques was anticipated, given that both are based on a kernel classifier (SVM). However, we remark that Kiedra provides additional valuable information about possible dependencies in the variable subsets, which wKiera do not. On the other hand, EBNA outperformed the kernel-based techniques in two cases (Horse–colic and Annealing), suggesting the Naive Bayes classifier may yield more effective discriminants for those datasets, although using larger feature subsets (almost twice the size).

**Table 4:** Average prediction accuracy in each dataset

Dataset	EBNA	wKiera	Kiedra
Ionosphere	92.40±2.04	98.07±1.36	98.49±0.9
Soybean	83.93±1.58	90.31±1.94	89.89±2.31
Horse–colic	88.64±1.70	82.31±2.78	81.42±3.81
Annealing	94.10±3.0	76.3±3.76	76.42±4.49
Image	88.98±0.98	89.55±1.28	90.29±1.78

Lastly, we also report on runtime performance statistics for the kernel–based methods (see Table 5). In average, wKiera needed fewer evaluations of the cost function in order to converge, compared to Kiedra; in terms of execution times there is no conclusive evidence of one method being faster that the other. However, we reckon these differences as being not remarkable, considering that Kiedra simultaneously produces estimates about possible variable dependencies. Unfortunately runtime information was not reported in the referenced EBNA report.

**Table 5:** Average runtime performance (only available for wKiera and Kiedra)

Dataset	wKiera		Kiedra	
	Evaluations	Time (secs.)	Evaluations	Time (secs.)
Ionosphere	2893.8±331.59	93.11±30.21	3009.00±838.81	87.62±36.06
Horse colic	1826.20±251.74	43.02±13.19	2258.20±507.71	75.07±25.68
Soybean-large	3501.00±437.77	151.04±38.42	4747.00±1647.87	141.34±48.16
Annealing	1059.40±159.38	87.76±15.42	1160.20±204.92	67.41±14.11
Image	1246.60±299.86	31.14±9.15	1541.80±860.79	44.76±24.40

### Experiment 3: Relevancy and dependency estimation on a medical domain

**Fig. 7:**
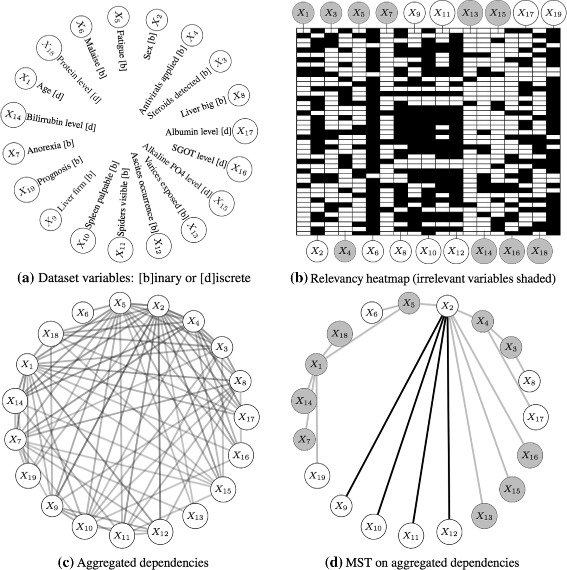
Relevancy and dependency discovery in the *Hepatitis* dataset using Kiedra (see the discussion of these results in the text). **a** Description of the variables in the dataset. **b** A heatmap of the discovered relevancy factors; each row correspond to the factors comprising the best solution of one repetition of the experiment. Relevant (*white*) and irrelevant (*shaded*) variables where chosen with a cutoff value of 0.5 on the average factors over all repetitions (only repetitions obtaining a classification accuracy greater than 80% were considered). **c** A graph of aggregated dependencies obtained over all repetitions. Opacity indicates the frequency of repetition a dependency was found. **d** The minimum-spanning-tree on the aggregated graph, suggesting the final estimated dependencies. Relevancy is also shown (irrelevant variables and associated dependencies are shaded)

The third study was focused on assessing the simultanoeus relevancy/dependency ability of the method on a medical domain. It was conducted on the *Hepatitis* dataset from the UCI repository [36, 37]. In this dataset, 19 clinical observations from 155 patients suffering from hepatitis were recorded, along with the final outcome (die or survive). The notation and domain of the variables in the dataset are given in Fig. 7(a).

The experiment was implemented using the same Kiedra testbed of Fig. 4; we simply changed the data source. The data was preprocessed and sampled for training and testing subsets as before. This protocol was repeated 100 times in order to prevent biased results due to randomness in the proposed method. Relevancy factors of the best solution found in each repetition were collected; then, variables discovered in more than half of the repetitions yielding accuracies greater than 80%, were selected as relevant (see the relevancy heatmap of Fig. 7(b)).

Additional variability was induced by shuffling the order of the patients in the dataset in each repetition. As a result we noticed dissimilar dependency trees were found. Thus, these trees were aggregated into a single graph, accounting the strengths of the dependencies as proportional to the number of times they showed up during the repetitions (see Fig. 7(c)). Lastly, in order to estimate the final pairwise dependencies, we computed the *minimum-spanning-tree* on this aggregated graph, using the inverse of the counts as edge costs and applying Kruskal’s algorithm [38] (see Fig. 7(d)).

Kiedra found a total of nine relevant variables: Sex (*X*_2_), Malaise (*X*_6_), Liver big (*X*_8_), Liver firm (*X*_9_), Spleen palpable (*X*_10_), Spiders visible (*X*_11_), Ascites occurrence (*X*_12_), Albumin level (*X*_17_) and Prognosis (*X*_19_). Besides, according to Fig. 7(b), the method found strong evidence of relevancy in subset {*X*_6_,*X*_19_}, followed by fair evidence of relevancy in {*X*_8_,*X*_9_,*X*_10_,*X*_11_,*X*_12_}, and lastly, borderline evidence in {*X*_2_,*X*_17_}. The first subset indicates, not surprisingly, that prognosis by histology is probably the most effective predictor of the disease, although being expensive and risky of complications [39]; similarly, malaise is seemingly correlative with the disease and it is a symptom usually reported by patients [40].

In contrast, the second subset correspond to more disease-specific symptoms: hepatomegaly (liver oversizing and stiffness) and splenomegaly (spleen enlargement) commonly reflect severity of liver damage [39, 41], spider nevi are visible in patients with the different variants of the disease [42, 43], and ascites has been reported as being strongly associated with hepatic dysfunctions [44]. Regarding the last subset, albumin is a protein synthesised in the liver, so it is reasonable to correlate changes in its level with infection with hepatitis. The peculiar finding here is Sex (*X*_2_), a non-disease-specific variable that nonetheless, has been recently linked to treatment response and survival rates with other unexpected features such as race (female, white) [39, 44]. In addition, it is worth noting that the relevant dependencies found by our method are between this variable and the other disease-specific *X*_9_,*X*_10_,*X*_11_,*X*_12_ predictors mentioned earlier.

We remark that these findings are corroborated by other related studies, such as [45] suggesting that variables *X*_19_, *X*_11_, *X*_17_ and *X*_12_ were highly indicative of the diagnosis. Likewise, [46] applied a method that discovered the subset of variables {*X*_6_,*X*_17_,*X*_14_,*X*_19_,*X*_11_} as relevant, with further experimentation finding predictive value in variable *X*_2_. Other studies using information theoretic, statistical and regularisation learning methods [47] as well as various machine learning and bioinspired techniques [48] also reported these variables in their relevant subsets, or report subsets with similar sizes (10–12 variables) obtaining similar prediction accuracies between 80–85% [49].

On the other hand, the following variables were characterised as not explanatory by Kiedra: Age (*X*_1_), Steroids detected (*X*_3_), Antivirals applied (*X*_4_), Fatigue (*X*_5_), Anorexia (*X*_7_), Varices exposed (*X*_13_), Bilirrubin level (*X*_14_), Alkaline PO4 level (*X*_15_), SGOT level (*X*_16_) and Protein level (*X*_18_). From this subset, it causes surprise bilirrubin not being discovered as indicative of the disease, as this protein is responsible of jaundice, the most common symptom related to fulminant hepatitis; we speculate that this may be due to the fact that bilirrubin levels differ depending on the type of illness and duration: acute or chronic, viral, drug–induced or autoimmune [44]. Unfortunately, in this dataset such information was not available. The other potential marker included in this subset is the alkaline phosphate level; however, some clinical studies have shown that this enzyme maintain normal levels during hepatitis infection, although it may raise in other hepatic–related injuries such as cholestasis [40].

No further evidence of other data mining studies assigning relevancy in the remainder variables was found [37, 47, 48]. Notice that consequently, we also regarded the dependencies associated with these variables as not relevant for the prediction of the disease outcome.

## Conclusion

We have described a method to tackle the dual combinatorial problem of relevancy-dependency discovery by coupling a weighted kernel classifier to guide the evolution of a probabilistic model of marginal and interacting effects among the problem features. Empirical evidence found in two experiments, one in a genetic epistasis testbed and another in a classification benchmark, indicates comparable performance with related baseline methods while providing richer dependency and relevancy information; in a third experiment comprising a hepatitis dataset, the method findings were corroborated with those reported in recent medical literature.

The promising potential mining capabilities of the method come at the expense of higher computational complexity of the algorithmic and data structures that it involves. In view of the nowadays increasingly availability of high–throughput and stream technologies for data acquisition, natural questions emerge in regards to the applicability of the method in large–scale scenarios. In this respect, we envisage two interesting avenues for further research, the first one related to algorithmic crafting so as to speed up the computation of the kernel function, which might be a bottleneck in such big data scenarios e.g. [14, 27, 50, 51]; the second one is considering compact representations of the probability model enabling memory and time savings during updating of its parameters [23, 52–54].

On a different perspective, the current design of the method is restricted to discrete probabilistic models; therefore modeling continuous distributions with its associated computational challenges, is also of significant interest. Besides, since the probabilistic model assumes a bivariate distribution, the method is prone to miss epistasis due to higher–order interactions, as it was shown in the hardest genetic problem in the empirical study. Thus, future work would also consider addressing this limitation.

As a final word, we also advocate adopting user–friendly visual graphical data–mining tools enabling biomedical analysts to focus on their experiments rather than on improving their low–level programming skills (see [55] for deeper insights on visual programming environments for bioinformatics). Hence, an additional challenge arising is growing and refining the suite of visual software components that currently implements the method.

## Additional file

Additional file 1 Additional Methods and Tools. (PDF 153 kb)
