# Supplementary material for: Discovering feature relevancy and dependency by kernel-guided probabilistic model-building evolution
Source: BioData Min. 2017 Mar 15;10:12. doi: 10.1186/s13040-017-0131-y (PMC5353680; doi:10.1186/s13040-017-0131-y)
Supplement: Additional file 1 — Additional Methods and Tools. (PDF 153 kb) [file 13040_2017_131_MOESM1_ESM.pdf]

METHODOLOGY

Discovering feature relevancy and dependency simultaneously using probabilistic model–building evolution and kernel machines

Nestor Rodriguez and Sergio Rojas–Galeano*

*Correspondence:
srojas@udistrital.edu.co.uk
Full list of author information is available at the end of the article

Additional Methods and Tools

Kiedra algorithm

The specification of `Kiedra` is shown in Algorithm 2. The algorithm adheres to the generic `EDA` template (Algorithm 1), with tailored sampling, selection and estimation steps so as to simultaneously discovering relevancy and dependency; its inputs are a labeled dataset, a kernel classifier instance including a parameterised kernel function (here the parameter p stands for the width σ of an RBF kernel or the degree d of a polynomial kernel; these options can be tuned empirically with respect to the input dataset prior to executing the algorithm). To begin with, the parameters of the probability model are initialised assuming an independent joint distribution (line 1) and the first pool of candidates is sampled from such distribution (line 2). Subsequent candidate pools are obtained by mixing samples from a bivariate binomial model with the current estimated parameters $\boldsymbol{\theta}$ (according to Equation (??)) and the most promising candidates kept from previous iterations (line 4). Then, roughly speaking, the algorithm iterates the evaluation of the candidates and re-estimation of the probability model until convergence.

Algorithm 2: `Kiedra`

Input: Dataset $D = \{(\mathbf{x}_i, y_i)\}_{i=1}^{N}$, algorithm $\mathcal{A}$, function $\kappa_p(\cdot,\cdot)$, pool size n

1 $\boldsymbol{\theta} \leftarrow \mathsf{initialise}(n)$
2 $\mathcal{B} \leftarrow \mathsf{sample}(P(X;\boldsymbol{\theta}), \frac{n}{2})$
3 **repeat** until $\boldsymbol{\theta}$ converges
4     $\mathcal{S} \leftarrow \mathsf{sample}(P(X;\boldsymbol{\theta}), \frac{n}{2}) \cup \mathcal{B}$; // According to Eq.(??)
5   **for each** $\mathbf{w}_k \in \mathcal{S}$ **do**
6         $\widetilde{D} \leftarrow D \otimes \mathbf{w}_k$
7         $f_k \leftarrow \mathsf{cross\_validation}(\mathcal{A}, \kappa_p(\cdot,\cdot), \widetilde{D})$; // According to Eq.(??) and (??)
8   **end**
9     $\mathcal{B} \leftarrow \mathsf{select}(\mathcal{S}, \{f_k\}, \frac{n}{2})$
10     $\mathcal{G}(\mathbf{V},\mathbf{E}) \leftarrow \mathsf{build\_graph}(\mathcal{B})$; // Using Eq.(??)
11     $\mathbf{E}_{\mathsf{MSF}} \leftarrow \mathsf{MSF}(\mathbf{E})$; // Using Eq.(??), (??) and (??)
12     $\boldsymbol{\theta} \leftarrow \mathsf{estimate}(\mathbf{E}_{\mathsf{MSF}}, \mathcal{B})$; // Using Eq.(??)
13     $\mathbf{b} \leftarrow \mathsf{update\_best}(\mathbf{b}, \mathcal{B}, \{f_k\})$
14 **end**

Output: Model parameters $\boldsymbol{\theta}$

The inner loop in line 8 is the core element of the algorithm, the one that evaluates the classification performance of the candidates, guiding the search for relevant variable subsets. This loop iterates over pool $\mathcal{S}$, plugging in each candidate as scale factors of the original dataset. The scaled dataset is then feed to the function $\kappa_p(\cdot, \cdot)$ according to Equation (**??**), and these kernel functions are used to train the kernel classifier $\mathcal{A}$ to learn the prediction rule of Equation (**??**). The actual fitness is obtained as the accuracy of $\mathcal{A}$ using a 5–fold cross–validation estimate of the classification accuracy.

The remaining steps execute the iterative updating of the dependency network and relevancy parameters. Firstly the subset of most promising candidates $\mathcal{B}$ are selected (line 9). Then, $\mathcal{B}$ is used to build the correlation graph (line 10) using Equation (**??**) , then the dependency forest using Equations (**??**), (**??**) and (**??**), and finally the new estimate for the parameters $\boldsymbol{\theta}$ is computed (line 12) using Equation (**??**). The last step keeps track of the best solution found up to that point (line 13).

Goldenberry suite of visual components

We used the `Orange` and `Goldenberry`[1] visual programming tools for data mining [1, 2, 3]. `Orange` provides a canvas where visual software components known as *widgets* can be wired together to execute several stages of a data mining task. `Goldenberry` is a add-on suite of widgets for stochastic–based search techniques (`EDA`, `GeneticAlgorithms`) and kernel classification machines (`Perceptron`, `SVM`); we actually extended this suite with new components so as to support `Kiedra` (e.g. `BMDA` and `FeatureSubset`). The `Kiedra` widgets were actually developed using the Python language and Numpy library for the logical modules, and the PyQt library for the graphical user interface. Additionally, in their implementation we employed multi-tasking concurrent techniques to speedup up execution of the main loop of the algorithm (fitness evaluation involving cross-validation of the classifier obtained with the relevance factors of each candidate from the pool $\mathcal{S}$ was run using multiple threads). A snapshot of the canvas and widgets used to perform the experiments can be seen in Figure 5.

The visual program that implements `wKiera` and `Kiedra` is shown in Figure 4. The key element here is the `WrapperCostFunction` widget which is wired to two input components: a `Data` widget that reads from the original dataset files and shuffles it randomly to provide training and testing subsets, and a `LearnerFactory`, which instantiates a kernel classifier and is configured with an appropriate tuned kernel function; in our experiments we chose the refined version of the `SVM` widget provided by `Goldenberry`, since it allows the definition of customised kernel functions and the creation of multiple classifier instances that can be executed in parallel. Other parameters such as cross-validation number of folds and accuracy/subset–size trade-off are also configured in the `WrapperCostFunction` widget, which takes a `BMDA` optimiser (top of the figure) to perform the search and estimation of relevancy and dependency following Algorithm 2. We observe that `wKiera` can be implemented almost identically, as it only requires to replace the optimiser by the

[1] `Orange` v3.3.1 is freely available from: http://orange.biolab.si; `Goldenberry` v2.0 is freely available from: http://goldenberry-labs.org (both last visited: July 14/2016).

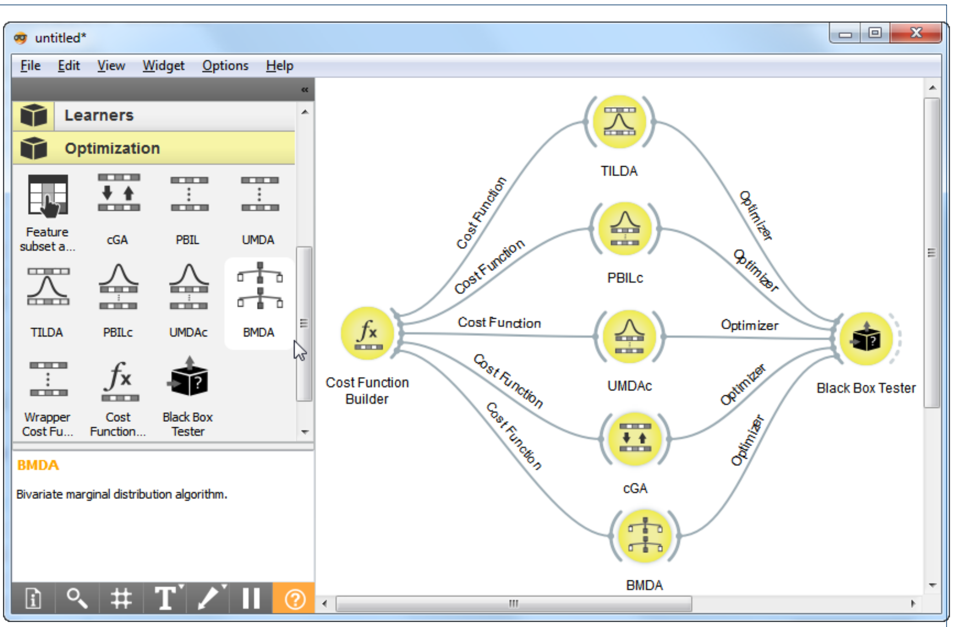

Figure 5 A `Goldenberry` program in the `Orange` canvas. In this program multiple `EDA`s are deployed to optimise a problem specified by the `CostFunctionBuilder` widget; the results obtained by each method are collected by the `BlackBoxTester` widget. The new `BMDA` widget developed for this study is highlighted in the library tab to the left and used at the bottom of the visual program.

`UMDA` widget (bottom of the figure); recall the latter assumes an independent probability model as thus provides only information about the relevancy of the input variables. Functionality description of these widgets are given in Table 1.

Table 1 List of `Orange` and `Goldenberry` widgets used in the experiments.

Widget	Purpose
`File`	Load input data from a text file.
`Preprocessing`	Fill missing values using a Naive Bayes classifier.
`Continuise`	Normalize data within the $[0,1]$ real interval.
`Datasampler`	Shuffle data and split into training and testing subsets.
`SVM`	Kernel machine whose kernel parameters are adjusted automatically for the input data by means of a grid–search.
`WrapperCostFunction`	Trades-off the simultaneous optimisation of accuracy vs. variable subset size (a weighted average with a contribution of 90% from accuracy). It also coordinates the m–fold cross–validation scheme ($m = 5$).
`BMDA`	The modified version of `BMDA` tailored for `Kiedra` (see Section ??). The pool size was set to four times the number of input variables.
`UMDA`	The `EDA` used in `wKiera`. The pool size again was set to four times the number of input variables
`BlackBoxTester`	The component that executes the experiments and collects statistics (10 repetitions were set per experiment).

Author details

References

1. Demšar, J., Curk, T., Erjavec, A., Črt Gorup, Hočevar, T., Milutinovič, M., Možina, M., Polajnar, M., Toplak, M., Starič, A., Štajdohar, M., Umek, L., Žagar, L., Žbontar, J., Žitnik, M., Zupan, B.: Orange: Data mining toolbox in python. Journal of Machine Learning Research **14**, 2349–2353 (2013)
2. Rojas-Galeano, S., Rodriguez, N.: Goldenberry: EDA visual programming in Orange. In: Proceedings of GECCO 2013, pp. 281–288. ACM, NY, USA (2013)
3. Garzón-Rodriguez, L.P., Diosa, H.A., Rojas-Galeano, S.: Deconstructing GAs into Visual Software Components. In: Proceedings of the Companion Publication of the 2015 on Genetic and Evolutionary Computation Conference. GECCO Companion '15, pp. 1125–1132. ACM, New York, NY, USA (2015)
